# Perspectives of nanotoxicology: Introduction

**DOI:** 10.1002/wnan.1843

**Published:** 2022-11-23

**Authors:** Nancy A. Monteiro‐Riviere

**Affiliations:** ^1^ Distinguished Professor Emeritus‐Regents & University Distinguished Professor of Toxicology Former Director, Nanotechnology Innovation Center of Kansas State (NICKS), Kansas State University Manhattan Kansas United States; ^2^ Professor Emeritus of Investigative Dermatology and Toxicology Center for Chemical Toxicology Research, North Carolina State University Raleigh North Carolina United States

The set of nano‐scale technologies applied to medicine today is broad and contains products and techniques which are at various stages of development. Public awareness and acceptance of nanomedicines is growing due to the development of safe and effective lipid nanoparticles mRNA COVID vaccines. In “early” days when researchers first studied nanomedicines, they did not have a good understanding of the unique mechanisms of nanomaterial toxicity and what safety biomarkers and endpoints were relevant to the development of safe nanomedicines. This state of affairs has now changed; it is well‐accepted that to obtain accurate results, a multitude of tests must be performed in order to assess the safety of any nanomedicines under development and to ensure that novel mechanisms of toxicity have been properly defined.

It is the goal of this review issue focused on nanotoxicology, to get a perspective of different facets of this topic from a broad international cohort of scholars active in this field. It is not a textbook of nanotoxicology and does not attempt to comprehensively cover all areas that could fall under this large umbrella. Instead, it focuses on topics that are relevant to the type of nanomedicines researchers and scientists are working on in the field today. The area of nanosafety assessment has been a concerted effort among government regulators, industrial sponsors, academicians, and the public to determine the potential hazards of nanotechnology.

This Nanotoxicology specialty issue begins with an overview “Toxicokinetics, dose‐response and risk assessment of nanomaterials: methodology, challenges, and future perspectives” of the methods used to conduct toxicokinetic, hazard identification, dose–response, exposure, and risk‐assessment of nanomaterials. It specifically focuses on the experimental design of plasma and tissue toxicokinetic studies and the use of physiological based pharmacokinetic (PBPK) models as an overarching tool to make *in vitro* to *in vivo* as well as animal to human extrapolations accurate.

A primary system that results in adverse events after exposure to nanomaterials is the immune system of man and animals. In the second manuscript “Understanding the immunological interactions of engineered nanomaterials: role of the bio‐corona”, the concept of synthetic versus biological identity of nanomaterials are discussed relative to the general properties that result in their interaction with the immune system. The role of how innate immunity classifies a nanomaterial as foreign and subsequently launches a protective response are presented. This topic continues into the next paper “Immunotoxicity of nanomaterials in health and disease: current challenges and emerging approaches for identifying immune modifiers in susceptible populations” which overviews the field of immunotoxicology and introduces the concept of adverse outcome pathways from the perspective of immune modulation and activation resulting in proinflammatory versus tolerogenic responses. The approach of integrating *in vitro* assays with *in silico* models to create comprehensive annotated data repositories is discussed. The next research topic “The importance of the IL‐1 family of cytokines in nanotoxicology and nano‐immunosafety” delves deeper into the immune response against nanomaterials by focusing on one specific family of immune mediators, the interleukin (IL)‐1cytokines. The potential mechanisms by which nanomaterials can cause IL‐1 cytokine release are reviewed.

The next topic “Nanoparticles for vaccine and gene therapy: Overcoming the barriers to nucleic acid delivery” involves the use of nanoparticles for the development of vaccines and gene therapy. The use of nucleic acid as nanomedicines to modulate all aspects of cellular function or as therapy against viral or microbial pathogens are hindered by the lack of efficient targeted delivery methods. The approaches by which nano‐based drug carriers that can be developed and targeted for these purposes are discussed. In a similar vein, the next contribution “The critical role of epigenetic mechanisms involved in nanotoxicology”, overviews the field of nanomaterial interactions with epigenetics. Can nanomaterials cause toxicity through DNA methylation, or modulation of non‐coding RNA expression or histone modifications? As these mechanisms are discovered, approaches can be developed to both decrease these effects or to develop more precise biomarkers of epigenetic effects. The last paper on nanomaterial genetic interactions “Genotoxicity testing of nanomaterials” overviews the broad field of testing methods used to assess the genotoxicity of nanomaterials. After introducing the methods, their relative merits are discussed relative to their application to nanomaterials versus organic molecules and issues relating to the actual design, conduction, interpretation, and validation of these tests.

The next paper “Nanoparticle‐peptide conjugates for bacterial detection and neutralization: potential applications in diagnostics and therapy**”** examines the application of nanoparticles to the development of more effective antimicrobial therapeutics and diagnostics specifically related to their coupling with antimicrobial peptides. This paper is followed by “Predicting nanomaterials pulmonary toxicity in animals by cell culture models: achievements and perspectives” dealing with the development of *in vitro* toxicology assays for studying the pulmonary toxicity of nanomaterials. Different cell culturing approaches are provided for their ability to predict in vivo effects in the lung. The next contribution “Bismuth nanomaterials as contrast agents for radiography and computed tomography imaging and their quality/safety considerations” reviews the development, disposition and safety assessment of bismuth nanoparticles used as radiographic contrast agents. Approaches for targeting these nanoparticles for specific diagnostic endpoints or even for their use for delivery of therapeutic agents is presented, as well as the mechanism of and amelioration of toxicity to the kidney that is often associated with them. The next paper “The potential for nanomaterial toxicity affecting the male reproductive system” introduces the topic of nanomaterial induced toxicity to the male reproductive system. The pathogenesis of direct and indirect toxicity to the testis as well as inflammatory responses and hormonal effects are reviewed. The final paper “Market entry system considering the biosafety of nanomedical devices in China”, discusses the development and implementation of a regulatory framework for assessing nanomaterial toxicity for risk assessment necessary for market access of nanomedical devices in China.

In conclusion, this series of articles provides a snapshot of the state of the art of nanotoxicology as practiced today, both from the presentation of basic mechanisms of nanomaterial toxicity to the regulatory tests established to detect adverse events. A common thread in this field is how hypotheses and methods developed for small organic molecules may be applied to nanomaterials. Are the principles of nanotoxicology significantly different than those of classic chemical toxicology? Advantages of using nanomaterials as therapeutics are also highlighted in numerous contributions as the approaches to administer them safely are explored.

The field of nanotoxicology and nanomedicine continues to grow substantially. In the beginning, nanomaterial safety was a concern. Studies for the proper characterization, nomenclature standards, physicochemical characteristics of the nanoparticles are needed to determine their toxicity in the biological milieu. There is still an enormous need to continue to develop additional tools for *in vivo* and *in vitro* toxicology testing to assess the safety of nanomedicines and gain insights into the mechanisms of interactions of nanomaterials with the biological systems.

